# Diagnostic performance of a loop-mediated isothermal amplification test for the diagnosis of human and cat sporotrichosis in two highly endemic areas in Brazil: insights from a real-world, field-based evaluation

**DOI:** 10.1007/s42770-026-02121-5

**Published:** 2026-10-21

**Authors:** Rodrigo Almeida-Paes, Lívia Silveira Munhoz, Susana Ruiz Mendoza, Vanice Rodrigues Poester, Vanessa Brito de Souza Rabello, Helena Schirmer, Márlon Grégori Flôres Custódio, Vanessa Suñé Mattevi, Dayvison Francis Saraiva Freitas, Melissa Orzechowski Xavier, Rosely Maria Zancopé-Oliveira, Cecília Bittencourt Severo

**Affiliations:** 1 Fundação Oswaldo Cruz, Laboratório de Micologia, Instituto Nacional de Infectologia Evandro Chagas, Rio de Janeiro, RJ Brazil; 2 Faculdade de Medicina, Programa de Pós-Graduação em Ciências da Saúde, Laboratório de Micologia, Universidade Federal do Rio Grande, RS Rio Grande, Brazil; 3 Universidade Federal de Ciências da Saúde de Porto Alegre (UFCSPA), Porto Alegre, RS Brazil; 4 Vice-Direção de Pesquisa e Desenvolvimento Tecnológico, Fundação Oswaldo Cruz, Instituto Nacional de Infectologia Evandro Chagas, Rio de Janeiro, RJ Brazil; 5 Fundação Oswaldo Cruz, Laboratório de Pesquisa Clínica em Dermatologia Infecciosa, Instituto Nacional de Infectologia Evandro Chagas, Rio de Janeiro, RJ Brazil

## Abstract

Sporotrichosis is a subacute to chronic mycosis of major public health relevance in endemic regions, affecting both humans and animals, particularly cats. Rapid and accessible diagnostic tools are urgently needed to improve case detection and disease surveillance. Thus, this study aimed to validate a previously described loop-mediated isothermal amplification (LAMP) assay for the diagnosis of sporotrichosis in clinical samples from humans and cats obtained in two highly endemic areas in Brazil. We conducted an observational study including human patients and cats with suspected sporotrichosis. Analytical sensitivity and specificity were initially assessed using reference strains of *Sporothrix* spp. The diagnostic performance of the LAMP assay was then evaluated in clinical samples at different incubation times and compared with culture results. Sensitivity, specificity, and additional diagnostic parameters were calculated separately for human and cat cases. The LAMP assay was able to detect the three major *Sporothrix* species associated with sporotrichosis, with higher analytical sensitivity for *S. brasiliensis*. However, when applied to human clinical samples, the assay showed limited sensitivity and specificity, particularly at earlier incubation times, resulting in poor overall diagnostic performance. In contrast, performance in cat samples was comparatively higher but still affected by false-positive and false-negative results. Despite promising analytical characteristics and previous success in experimental models, this LAMP protocol demonstrated limited diagnostic utility for human and cat sporotrichosis under real-world clinical conditions, highlighting the need for further optimization and rigorous clinical validation before implementation in routine practice.

**Supplementary Information:**

The online version contains supplementary material available at https://doi.org/10.1007/s42770-026-02121-5.

## Introduction

Sporotrichosis is a subacute to chronic granulomatous mycosis caused by fungi of the *Sporothrix* genus, with *Sporothrix brasiliensis*, *Sporothrix globosa*, and *Sporothrix schenckii* being the most clinically relevant species globally [1]. This infection affects both humans and a variety of animal hosts, particularly domestic cats, which play a central role in zoonotic transmission through traumatic inoculation of fungal yeasts into the skin and subcutaneous tissues [2, 3]. In endemic regions, especially in Brazil, sporotrichosis has emerged as a notable public health concern due to its increasing incidence and the challenges associated with timely diagnosis and management [4, 5]. Conventional diagnostic approaches for both humans and cats rely heavily on fungal culture and histopathological evaluation, which, despite high specificity, are inherently slow and dependent on specialized laboratory infrastructure and technical expertise [1, 6].

Therefore, rapid and accurate detection of *Sporothrix* species is critical for guiding clinical decision-making and reducing transmission risk [7]. Molecular methods such as polymerase chain reaction (PCR) have been explored to overcome limitations of culture-based diagnostics, offering improved sensitivity and turnaround time [8–10]. However, PCR typically requires expensive equipment, controlled laboratory environments, and trained personnel, factors that may limit their implementation in resource-constrained settings or point-of-care contexts [11, 12].

Loop-mediated isothermal amplification (LAMP) has emerged as an attractive alternative for molecular diagnosis, combining high specificity and sensitivity with the ability to amplify target nucleic acids under isothermal conditions [13]. This characteristic obviates the need for thermocyclers and enables rapid amplification with minimal instrumentation, often allowing results to be visualized by simple colorimetric changes. Such features make LAMP particularly suitable for diagnostic applications, including on-site testing in veterinary clinics and clinics with limited laboratory capacity [14].

Recent studies have reported the development of *Sporothrix*-targeted LAMP assays that demonstrate promising analytic performance when applied to isolates and experimental samples. These assays, designed against conserved genetic loci such as the calmodulin gene or ribosomal RNA regions, have shown high specificity and low detection limits, underscoring the potential utility of LAMP for rapid screening of sporotrichosis [15–17]. Preliminary evaluations using animal models of experimental disease or samples from animals with known sporotrichosis status further suggest the feasibility of extending LAMP to real-world diagnostic workflows [16, 17]. Despite these advances, systematic validation of LAMP performance across diverse clinical sample types from both human and animal hosts remains limited, and standardized data on diagnostic accuracy, reproducibility, and applicability under field conditions are scarce.

In this study, we aimed to evaluate the diagnostic performance of a LAMP assay previously described by our group [16] when applied directly to clinical samples from humans and animals in endemic settings. By assessing analytical sensitivity, specificity, and diagnostic performance, our work seeks to evaluate whether the current LAMP protocol that our group developed can serve as a robust, rapid, and accessible diagnostic tool to support improved detection and surveillance of this neglected mycosis in both human and veterinary medicine.

## Materials and methods

### Study design

This was a prospective observational study conducted at two centers, using swab or skin tissue samples collected from cutaneous lesions of human and animal patients with suspected sporotrichosis. The centers are located in two major endemic areas for sporotrichosis in Brazil: the Evandro Chagas National Institute of Infectious Diseases (INI/Fiocruz), in Rio de Janeiro, and the Federal University of Rio Grande (FURG), in Rio Grande do Sul. Human patients were enrolled at both centers, whereas cats were included only at the Rio Grande do Sul center. Inclusion criteria for human patients comprised adult individuals (≥ 18 years old) with a clinical and epidemiological history suggestive of sporotrichosis whose biological samples, collected between 2023 and 2024, were consecutively referred to the Mycology Laboratories of the participating centers. The study protocol was approved by the institutional ethics committees (protocol number 59605222.7.0000.5262), and all participants provided written informed consent prior to sample collection. Inclusion criteria for cats included animals of any age presenting cutaneous lesions compatible with sporotrichosis. The use of animal samples was exempt from approval by the Ethics Committee on Animal Use from FURG, as all specimens were obtained during routine veterinary care and no procedures were performed specifically for research purposes or beyond standard clinical management.

### Case definitions

Case definitions were adapted from the European Organization for Research and Treatment of Cancer/Mycoses Study Group Education and Research Consortium (EORTC/MSGERC) and applied in accordance with the Standards for Reporting Diagnostic Accuracy Studies (STARD) guidelines. Proven sporotrichosis was defined as the isolation of *Sporothrix* spp. from a clinical specimen by fungal culture, followed by species identification using phenotypic and/or molecular methods, irrespective of clinical presentation. Culture positivity was considered the reference standard for definitive case classification. Probable sporotrichosis was defined by the presence of compatible clinical features (e.g., nodular, ulcerative, or lymphocutaneous lesions), relevant epidemiological exposure, and at least one of the following laboratory test results: (i) direct microscopic examination suggestive of *Sporothrix* spp.; (ii) histopathological findings consistent with sporotrichosis; (iii) detection of anti-*Sporothrix* IgG antibodies in serum; or (iv) detection of *Sporothrix* DNA by a non–culture-based molecular method. A favorable clinical response to antifungal therapy was considered a complementary criterion but was not used in isolation to define probable cases. These case definitions were applied uniformly to human and animal subjects to ensure standardized case classification and comparability of diagnostic performance analyses.

### Sample processing and DNA extraction

DNA from clinical specimens obtained from human and animal samples was extracted using the QIAamp DNA Mini Kit (QIAGEN, Hilden, Germany), according to the manufacturer’s instructions.

### LAMP conditions

LAMP reactions were performed as previously described by our group using the WarmStart^®^ Colorimetric LAMP 2× Master Mix (New England Biolabs, Ipswich, MA, USA) in a final volume of 25 µL [16]. Each reaction contained 12.5 µL of 2× Colorimetric LAMP Master Mix, 1.6 µM of each inner primer (FIP_SBRAZ: TCGGAGGGATTCTGGCCGAGAGGCCAGATCACCACCAA;BIP_SBRAZ:AGCTGCAGGACATGATCAACGAGGTCAAACGTACCAGGGAAG),0.2 µM of each outer primer (F3_SBRAZ: CAGTCGAACTGACGCTGTT; B3_SBRAZ: CGCATCATGCCACATTCAAC), 0.4 µM of each loop primer (LF_SBRAZ: ATGACGGTGCCGAGCT; and LB_SBRAZ: GGTCGACGCCGACAACA), and 2 µL of template DNA. Nuclease-free water was added to reach the final reaction volume. Amplification was carried out under isothermal conditions at 65 °C for 30, 40, 50–60 min. LAMP amplification was considered positive when a visible color change from pink to orange/yellow was observed, indicating successful DNA amplification. Each run included a positive control containing *S. brasiliensis* genomic DNA and a negative control containing nuclease-free water in place of template DNA to monitor assay performance and potential contaminations. All reactions were performed in duplicate, and results were independently assessed by two investigators blinded to the sample identity. Reaction tubes remained sealed after the addition of template DNA to prevent amplicon carryover contamination between samples.

### *In silico* evaluation of LAMP primer architecture and target coverage

The theoretical target coverage of the LAMP assay was investigated using a Python workflow developed for comprehensive in silico evaluation of LAMP primer architecture. The workflow integrates target retrieval, primer architecture reconstruction, mapping of individual primer-binding regions, mismatch analysis, and automated generation of summary tables and vector figures. First, the external primers F3_SBRAZ and B3_SBRAZ were queried against the NCBI nucleotide database using Primer-BLAST, restricting the search to *Sporothrix* calmodulin (CAL) sequences. Primer-BLAST output was parsed using LAMPMap to extract accession numbers, organism names, predicted amplicon lengths, primer sequences, primer coordinates, and F3/B3 amplification intervals. All nucleotide records retrieved by Primer-BLAST were then downloaded from GenBank using Entrez Direct, generating a local FASTA dataset for downstream analyses. The original reference sequence used for primer design, GenBank accession OM473285.1, was used to reconstruct the complete LAMP primer architecture. Because LAMP inner primers are composed of two independent target-binding regions, FIP_SBRAZ was evaluated as its constituent regions F1c and F2, and BIP_SBRAZ as B1c and B2. Together with the external primers F3/B3 and loop primers LF/LB, eight primer-binding regions were therefore evaluated: F3, F2, LF, F1c, B1c, LB, B2, and B3. Each primer-binding region was independently mapped against every retrieved CAL sequence in both forward and reverse-complement orientations. For each target sequence, LAMPMap recorded primer coordinates, strand orientation, number of matches, and whether each binding site was located within the predicted F3–B3 amplicon. Two complementary mapping strategies were performed. The first required exact nucleotide identity across each complete primer-binding region. The second allowed up to one nucleotide mismatch per primer-binding region, providing a permissive estimate of primer conservation among closely related *Sporothrix* sequences. For each accession, the workflow automatically determined the presence of external primers, FIP constituent regions, BIP constituent regions, loop primers, the complete LAMP architecture, and whether all primer-binding regions were located within the predicted amplification interval. Summary statistics were generated for the complete dataset and for species-specific subsets.

### *In vitro* evaluation of LAMP primers

Subsequently, genomic DNA from ten fungal strains; S. schenckii (IPEC 36277), S. brasiliensis (IPEC 50532), S. brasiliensis (IPEC 98 − 2), S. globosa (IPEC 271385), S. globosa (IPEC 34540), Histoplasma capsulatum (IPEC 09/12), Fonsecaea pedrosoi (IPEC 58771), Penicillium chermesinum (IPEC 6176 H), Aspergillus flavus (IPEC 56532), and Candida metapsilosis (VGC 104); was quantified using a NanoDrop™ 2000/2000c spectrophotometer. To determine the LAMP detection limit, six serial DNA dilutions (5, 2, 1, 0.2, 0.1, and 0.02 ng/µL) of Sporothrix spp. DNA were prepared and submitted to the LAMP reaction. To determine LAMP specificity, all DNA at 5 ng/µL were submitted to the LAMP reaction. Amplification was assessed by visual colorimetric reading and confirmed by electrophoresis on 2% agarose gels.

### Data analysis

Diagnostic performance of the LAMP assay was evaluated separately for human and animal samplesand according to the different incubation times assessed. Fungal culture was the reference standard for the diagnosisof sporotrichosis. LAMP results were classified as positive or negative based on the colorimetric change at eachpredefined time point, as previously explained. Sensitivity was calculated as the proportion of culture-positive samplesthat tested positive by LAMP, whereas specificity was calculated as the proportion of culture-negative samples thattested negative by the molecular test. The positive likelihood ratio (LR+) was calculated as sensitivity divided by (1− specificity), and the negative likelihood ratio (LR−) as (1 − sensitivity) divided by specificity. The diagnostic oddsratio (DOR) was calculated as the ratio of LR+ to LR−, equivalent to the odds of a positive test result among patientswith sporotrichosis relative to those without the disease. For each group (humans and animals) and for each incubationtime, sensitivity and specificity were expressed as proportions, with corresponding 95% confidence intervals (95%CIs). Discordant results between LAMP and culture were further examined descriptively. All analyses were conductedusing MedCalc Statistical Software version 23.0.8 (MedCalc Software Ltd, Ostend, Belgium; https://www.medcalc.org ;2024).

## Results

### *In silico *and experimental evaluation of LAMP primer performance

**Fig. 1:**
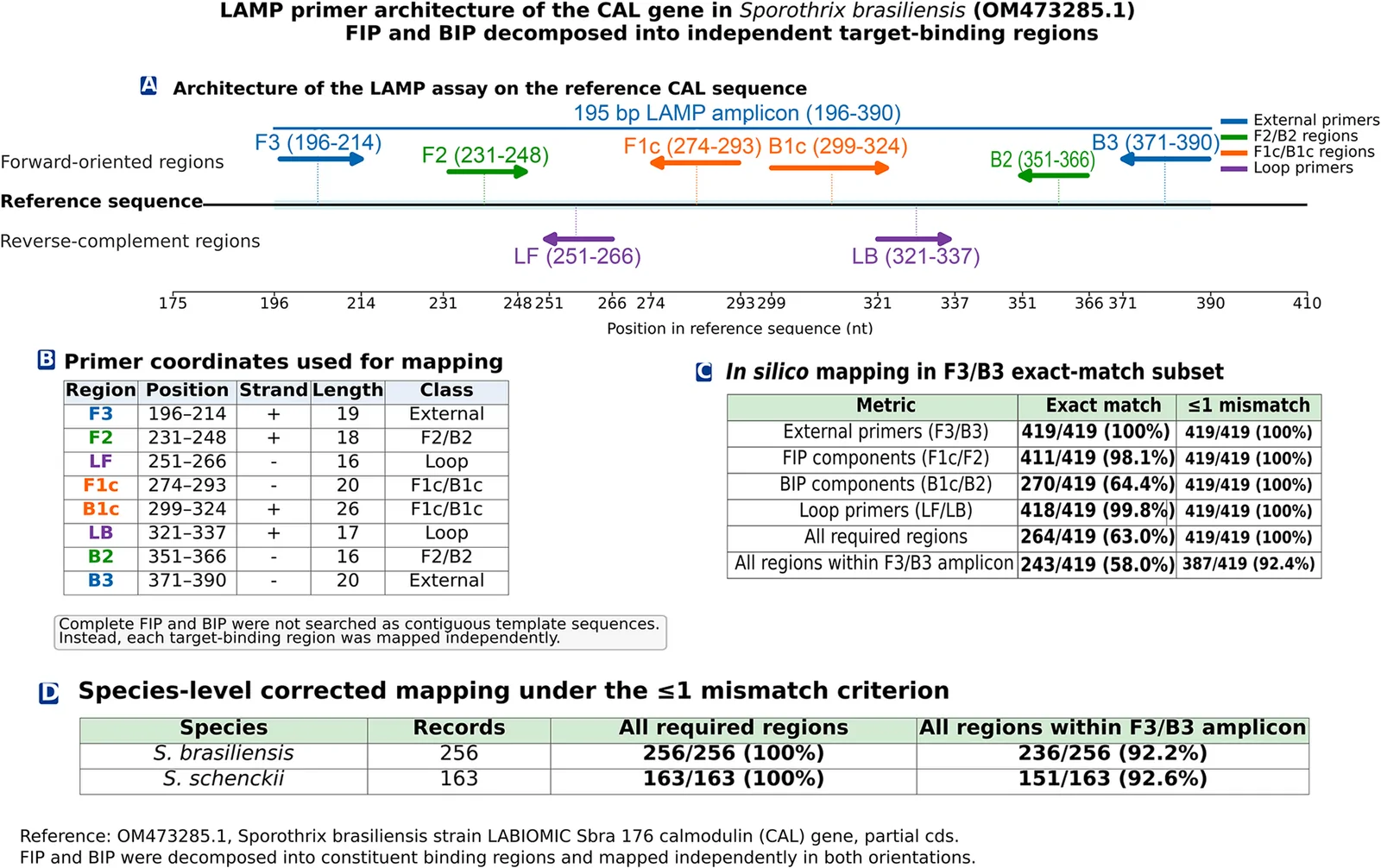
*In silico* characterization of the LAMP primer architecture targeting the calmodulin (CAL) gene of *Sporothrix*. **(A)** Architecture of the LAMP assay reconstructed on the *Sporothrix brasiliensis* reference CAL sequence (GenBank accession OM473285.1), showing the positions and orientations of the eight functional primer-binding regions within the 195-bp F3–B3 amplification interval. **(B)** Coordinates, strand orientation, length, and functional classification of each primer-binding region. FIP and BIP were decomposed into their constituent target-binding regions (F1c/F2 and B1c/B2, respectively), which were evaluated independently. **(C)** Summary of primer-binding region conservation among the 419 sequences exhibiting exact F3/B3 matches, comparing strict exact-match and ≤ 1 mismatch criteria. **(D)** Species-level conservation of the complete LAMP architecture under the ≤ 1 mismatch criterion for *S. brasiliensis* and *S. schenckii*

The in silico evaluation identified 792 Sporothrix sequences using the external primers F3_SBRAZ and B3_SBRAZ, of which 419 exhibited 100% identity with both external primers and were selected for detailed analysis (Supplementary Table S1). The complete LAMP primer architecture was reconstructed on the reference CAL sequence of S. brasiliensis (GenBank accession OM473285.1), defining a 195-bp F3–B3 amplification interval (Fig. 1A). The inner primers were evaluated according to their constituent target-binding regions, with FIP decomposed into F1c and F2 and BIP into B1c and B2. Together with the external primers (F3 and B3) and loop primers (LF and LB), eight functional primer-binding regions were independently evaluated in both orientations (Fig. 1B). Within the 419-sequence F3/B3 exact-match subset, the external primers were conserved in all sequences (419/419; 100%). Exact matches for both FIP components were detected in 411/419 sequences (98.1%), whereas both BIP components were detected in 270/419 sequences (64.4%). The loop primer regions were highly conserved, with both LF and LB detected in 418/419 sequences (99.8%). Overall, all eight required primer-binding regions were detected by exact matching in 264/419 sequences (63.0%), with 243/419 (58.0%) retaining the complete architecture within the predicted F3–B3 amplicon (Fig. 1C; Supplementary Table S2). When up to one nucleotide mismatch per primer-binding region was allowed, all eight required regions were detected in all 419 sequences (100%), and 387/419 sequences (92.4%) retained the complete architecture within the F3–B3 amplification interval (Fig. 1C; Supplementary Table S2). At the species level, all required primer-binding regions were detected under the ≤ 1 mismatch criterion in both S. brasiliensis (256/256; 100%) and S. schenckii (163/163; 100%). Complete architecture within the predicted amplicon was observed in 236/256 (92.2%) S. brasiliensis sequences and 151/163 (92.6%) S. schenckii sequences (Fig. 1D).

**Fig. 2:**
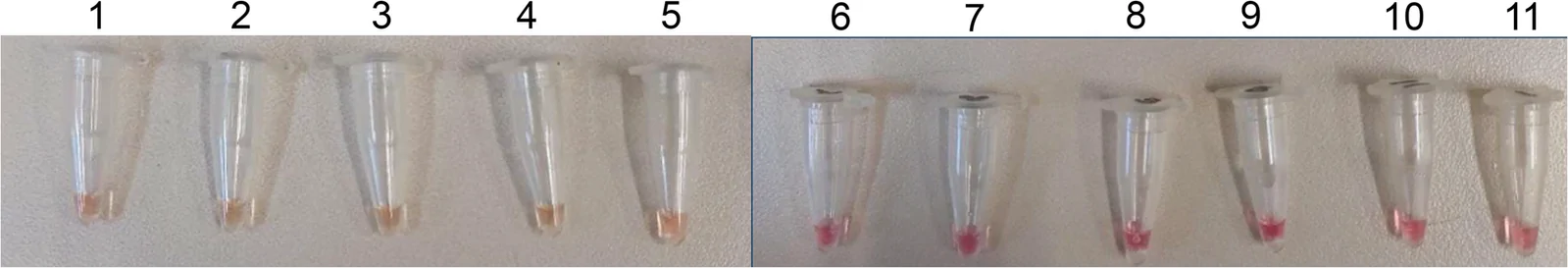
LAMP reactions with three *Sporothrix* species *(S. brasiliensis* – tubes 2 and 3, *S. globosa* – tubes 4 and 5, and *S. schenckii* – tube 1) and five unrelated fungal species (*Histoplasma capsulatum* – tube 6, *Fonsecaea pedrosoi* – tube 7, *Penicillium chermesinum* – tube 8, *Aspergillus flavus* – tube 9, and *Candida metapsilosis –* tube 10. Tube 11 consists of a negative control (no fungal DNA)

These findings were further confirmed by experimental assays, which showed that the LAMP assay could detect the three major *Sporothrix* species associated with sporotrichosis, with a detection limit of 200 pg/µL of genomic DNA for *S. schenckii* and *S. globosa*, and higher analytical sensitivity for *S. brasiliensis*, with a detection limit of 20 pg/µL. Other species tested were negative in the LAMP reaction (Fig. 2).

### LAMP diagnostic performance for human sporotrichosis

The two participating centers enrolled a total of 76 human patients: 40 from Rio de Janeiro and 36 from Rio Grande do Sul. Among these patients, 35 were classified as having proven sporotrichosis (22 from Rio de Janeiro and 13 from Rio Grande do Sul), 8 as probable sporotrichosis (6 from Rio de Janeiro and 2 from Rio Grande do Sul), and 33 received alternative diagnoses (e.g., tuberculosis, leishmaniasis, chromoblastomycosis, paracoccidioidomycosis, pyodermitis) or were referred to other healthcare facilities without a definitive diagnosis (12 from Rio de Janeiro and 21 from Rio Grande do Sul). A total of 76 clinical specimens were analyzed, comprising 64 abscess swabs and 12 skin biopsy fragments. LAMP results were assessed at four predefined incubation times by two investigators blinded to the sample identity, with 100% concordance at both centers. At 30 and 40 min, only one patient with proven sporotrichosis tested positive. At 50 min, four patients, all from Rio Grande do Sul, tested positive, including two patients with proven sporotrichosis and two patients without a sporotrichosis diagnosis. At 60 min, 33 patients (6 from Rio Grande do Sul and 27 from Rio de Janeiro) tested positive, including 18 patients with proven sporotrichosis, three with probable sporotrichosis, and 12 patients without sporotrichosis diagnosed by other methods. Table 1 summarizes the sensitivity, specificity, and additional diagnostic performance parameters of the LAMP assay for the diagnosis of human sporotrichosis at both centers. We did not observe significant differences among LAMP performance using different clinical samples, with false positive and false negative results on both specimens. Among patients with positive LAMP results who received alternative infectious disease diagnoses based on their respective reference standard methods, positive LAMP results were observed in cases of tuberculosis (n = 2) and chromoblastomycosis (n = 2), all of them from RJ. In addition, four patients classified as having probable sporotrichosis also yielded positive LAMP results. Among patients classified as having probable sporotrichosis who had negative LAMP results, two yielded positive PCR results for Sporothrix spp. Conversely, among patients with culture-confirmed Sporothrix spp. infection who tested negative by LAMP, all three major clinical presentations of sporotrichosis were represented, including lymphocutaneous, fixed cutaneous, and disseminated cutaneous forms.

**Table 1:** Diagnostic performance of a calmodulin-based Loop-Mediated Isothermal Amplification (LAMP) assay for human sporotrichosis at different reading times, with corresponding 95% confidence intervals

Center	Parameter	LAMP reading time (minutes)			
		30	40	50	60
RJ^a^	Sensitivity	0 (0–0.15)	0 (0–0.15)	0 (0–0.15)	0.73 (0.50–0.89)
	Specificity	1 (0.81–1)	1 (0.81–1)	1 (0.81–1)	0.39 (0.17–0.64)
	PLR^c^	ND^e^	ND	ND	1.19 (0.76–1.86)
	NLR^d^	1	1	1	0.70 (0.29–1.72)
RS^b^	Sensitivity	0.08 (0.001–0.36)	0.08 (0.001–0.36)	0.15 (0.02–0.45)	0.15 (0.02–0.45)
	Specificity	1 (0.85–1)	1 (0.85–1)	0.91 (0.72–0.99)	0.83 (0.61–0.95)
	PLR	ND	ND	1.77 (0.28–11.12)	0.88 (0.19–4.19)
	NLR	0.92 (0.79–1.08)	0.92 (0.79–1.08)	0.93 (0.71–1.21)	1.02 (0.76–1.38)
Both	Sensitivity	0.02 (0.0007–0.15)	0.02 (0.0007–0.15)	0.05 (0.007–0.19)	0.51 (0.34–0.69)
	Specificity	1 (0.91–1)	1 (0.91–1)	0.95 (0.83–0.99)	0.63 (0.47–0.78)
	PLR	ND	ND	1.17 (0.17–7.89)	1.41 (0.84–2.35)
	NLR	0.97 (0.92–1.03)	0.97 (0.92–1.03)	0.99 (0.89–1.10)	0.77 (0.51–1.16)

^a^ RJ: Rio de Janeiro

^b^ RS: Rio Grande do Sul

^c^ PLR: positive likelihood ratio

^d^ NLR: negative likelihood ratio

^e^ ND: not determined. No positive results in the assay

#### LAMP diagnostic performance in humans using different clinical samples

Among the 40 patients enrolled in Rio de Janeiro, 12 provided skin biopsy specimens and 28 provided lesion swab samples. In contrast, all patients enrolled in Rio Grande do Sul were sampled exclusively by lesion swab. Given the marked differences in LAMP sensitivity and specificity observed between the two centers (Table 1), the comparison of assay performance according to clinical specimen type was restricted to the Rio de Janeiro cohort. Because no patient from Rio de Janeiro tested positive before 60 min of incubation, this analysis was performed using LAMP results obtained after 60 min. The diagnostic performance of the assay according to specimen type is presented in Table 2. Although lesion swabs yielded slightly higher sensitivity (75% vs. 67%) and positive likelihood ratios (1.29 vs. 1.00) than tissue biopsy specimens, the wide and overlapping 95% confidence intervals indicate no clear evidence of superior diagnostic performance for either specimen type.

**Table 2:** Diagnostic performance of a calmodulin-based loop-mediated isothermal amplification (LAMP) assay for the diagnosis of human sporotrichosis using different clinical specimen types at a 60-minute incubation time, with corresponding 95% confidence intervals

Parameter	Skin Biopsy Fragment	Abscess Swab
Sensitivity Specificity Positive Likelihood Ratio Negative Likelihood Ratio	0.67 (0.22–0.96) 0.33 (0.043–0.78) 1.00 (0.45–2.23) 1.00 (0.20–4.95)	0.75 (0.48–0.93) 0.42 (0.15–0.72) 1.29 (0.74–2.24) 0.60 (0.20–1.77)

### LAMP diagnostic performance for cat sporotrichosis

A total of 90 cats were evaluated at FURG, including 51 classified as having proven sporotrichosis, four as probable sporotrichosis, and 35 without a sporotrichosis diagnosis based on other diagnostic methods. LAMP results were assessed at predefined incubation times. After 30 min, positive LAMP results were observed in 21 cats, all of which had proven sporotrichosis. At 40 min, 30 cats yielded positive results, including 28 cats with proven sporotrichosis and two cats without a sporotrichosis diagnosis. After 50 min, 33 cats tested positive, including 30 with proven sporotrichosis and three without a sporotrichosis diagnosis. At 60 min, 41 cats yielded positive LAMP results, including 33 cats with proven sporotrichosis, two cats with probable sporotrichosis and six cats without a sporotrichosis diagnosis. Table 3 summarizes the sensitivity, specificity, and additional diagnostic performance parameters of the LAMP assay for the diagnosis of cat sporotrichosis.

**Table 3:** Diagnostic performance of a calmodulin-based Loop-Mediated Isothermal Amplification (LAMP) assay for cat sporotrichosis at different reading times, with corresponding 95% confidence intervals

Parameter	LAMP reading time (minutes)			
	30	40	50	60
Sensitivity	0.41 (0.28–0.56)	0.55 (0.40–0.69)	0.59 (0.44–0.72)	0.65 (0.50–0.78)
Specificity	1 (0.91–1)	0.95 (0.83–0.99)	0.92 (0.79–0.98)	0.79 (0.64–0.91)
PLR^a^	ND^c^	10.71 (2.71–42.24)	7.65 (2.52–23.23)	3.15 (1.65–6.04)
NLR^b^	0.59 (0.47–0.74)	0.48 (0.35–0.65)	0.45 (0.32–0.63)	0.44 (0.30–0.67)

^a^ PLR: positive likelihood ratio

^b^ NLR: negative likelihood ratio

^c^ ND: not determined. No false-positive results in the assay

### Comparison of LAMP performance in cat and human populations

**Fig. 3:**
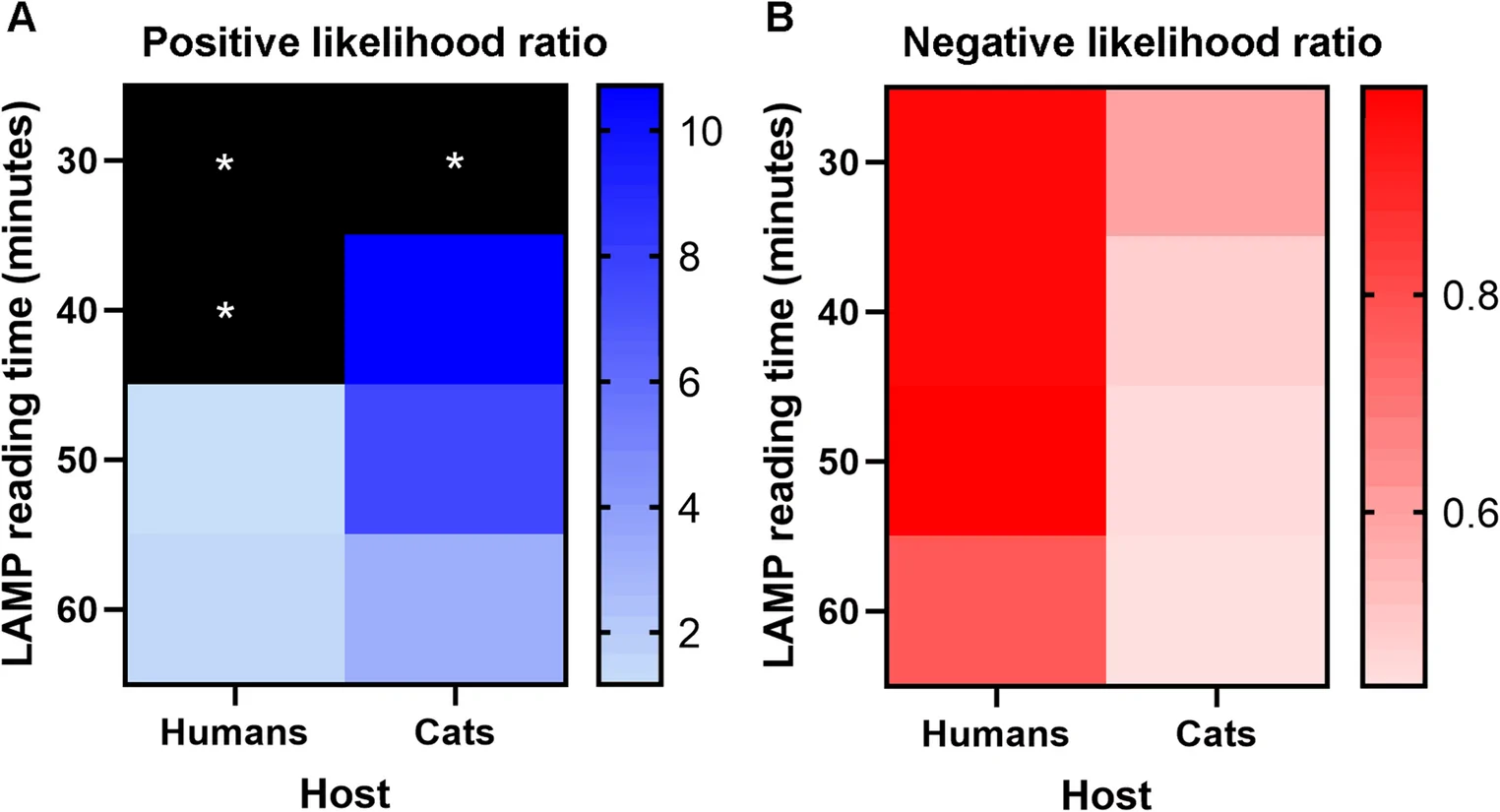
Heatmap of positive (**A**) and negative (**B**) likelihood ratios of the Loop-Mediated Isothermal Amplification (LAMP) assay in human and cat samples across different incubation times. Color intensity represents the magnitude of likelihood ratios, with higher positive likelihood ratios indicating stronger rule-in capacity and lower negative likelihood ratios indicating stronger rule-out capacity. Cells corresponding to time points with 100% specificity and no false-positive results are indicated with *

**Fig. 4:**
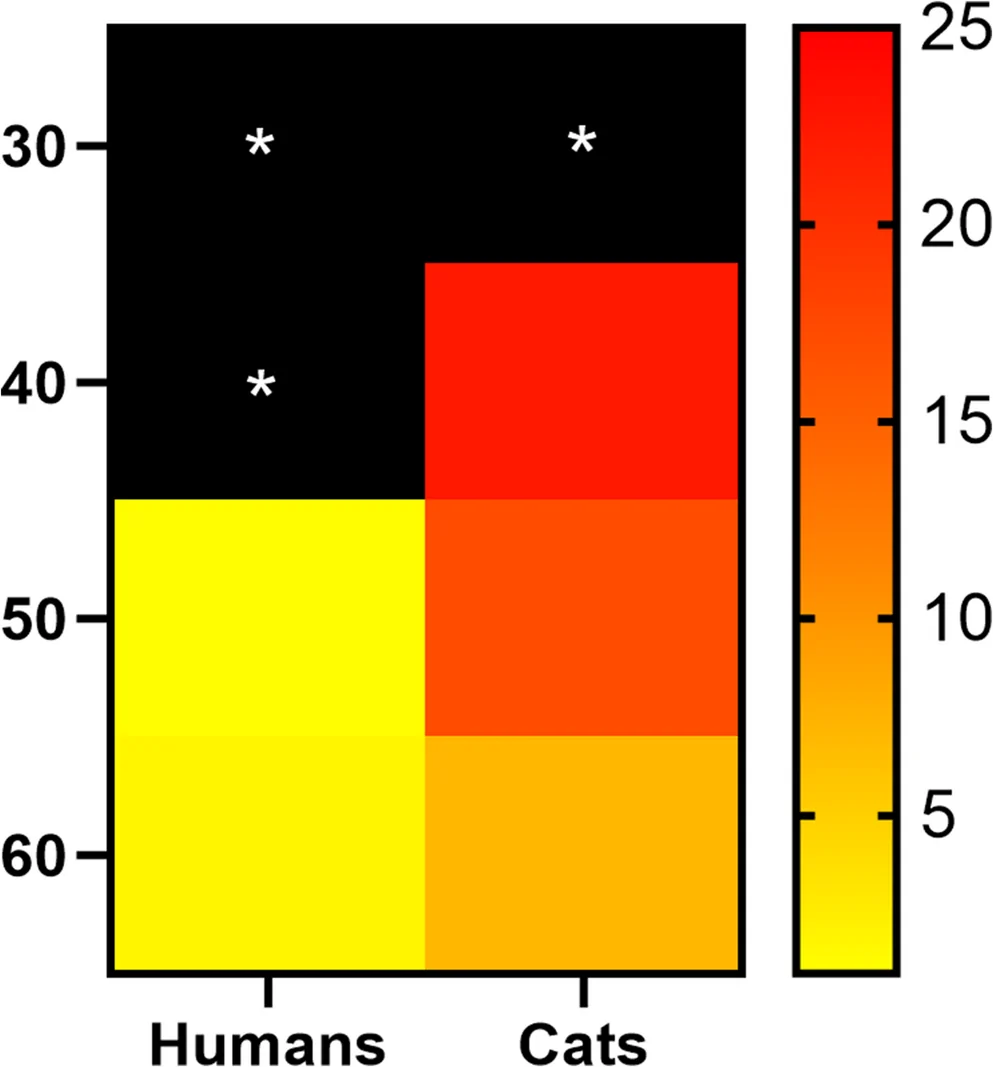
Heatmap of diagnostic odds ratios of the Loop-Mediated Isothermal Amplification (LAMP) assay in human and cat samples across different incubation times. Color intensity represents the magnitude of diagnostic odds ratios, with higher ratios indicating a better and more accurate test. Cells corresponding to time points at which no false-positive results were observed and specificity was therefore 100% are indicated with an asterisk (*). At these time points, the positive likelihood ratio and DOR were mathematically undefined and are reported as not estimable because their calculation involves division by zero

Figure 3 compares the likelihood ratios of the LAMP assay in human and cat samples across the different incubation times evaluated. In humans at 30 and 40 min, and in cats at 30 min, no false-positive results were observed. Consequently, specificity reached 100%, and the positive likelihood ratio could not be calculated, being mathematically undefined and theoretically infinite. At the remaining time points, positive likelihood ratios were consistently higher in cats than in humans, indicating a stronger rule-in capacity of the assay in the cat population. Figure 4 presents the diagnostic odds ratios (DORs) of the LAMP assay in human and feline samples at the different incubation times evaluated. In humans, the low DORs obtained after 50 and 60 min of incubation reflect the limited ability of the assay to discriminate between sporotrichosis and non-sporotrichosis cases, reinforcing its poor diagnostic performance in this population. In contrast, the highest DOR (22.52) was observed in feline samples after 40 min of incubation, suggesting that this incubation time provides the most favorable balance between sensitivity and specificity.

## Discussion

In this study, we conducted a real-world evaluation of a colorimetric LAMP assay for the diagnosis of sporotrichosis in human and cat clinical samples obtained from two highly endemic areas in Brazil. Although previous experimental and animal model studies conducted by our group demonstrated promising performance of this assay under controlled laboratory conditions [16], the present findings reveal important limitations, particularly with respect to its diagnostic value for human sporotrichosis in routine clinical settings.

This study used human and cat clinical samples collected in two highly endemic regions of Brazil [5, 18], representing a major strength by reflecting real-world diagnostic conditions. The use of standardized case definitions, culture as the reference standard, and assessment across multiple incubation times further support the robustness of the analysis. However, important limitations include the heterogeneity of clinical samples, potential variability in sampling quality, and the evaluation of alternative DNA extraction methods [19, 20], which may have affected diagnostic performance, particularly in human cases. In fact, all samples from Rio Grande do Sul were collected by swabs, which may carry less DNA than biopsy samples. Because lesion swabs represent the primary specimen routinely collected for diagnostic purposes [1], only a small number of patients underwent skin biopsy. Although the results obtained with the two specimen types appeared broadly comparable in the Rio de Janeiro cohort, the limited number of biopsy samples precludes definitive conclusions regarding their relative diagnostic performance.

A striking observation of the study was the modest diagnostic performance of the LAMP assay in human samples, especially at earlier incubation times. At 30 and 40 min, LAMP detected only a single culture-confirmed human case, resulting in unacceptably low sensitivity for clinical use. Even at 60 min, the incubation time associated with the highest number of positive results, LAMP positivity was observed in a substantial proportion of patients without sporotrichosis diagnosis based on reference methods, indicating a loss of specificity. The likelihood ratio and diagnostic odds ratio analyses corroborated the findings obtained from sensitivity and specificity estimates. Whereas LR+ values were consistently higher and LR− values lower in feline samples than in human samples, indicating superior rule-in and overall diagnostic performance, none of the evaluated incubation times produced LR− values compatible with confidently excluding sporotrichosis. Likewise, the highest diagnostic odds ratio was observed in feline samples after 40 min of incubation, suggesting that this time point provided the best balance between sensitivity and specificity. Conversely, the low diagnostic odds ratios observed in human samples, particularly after 50 and 60 min, indicate poor discriminatory ability.

Although laboratory procedures were fully standardized across both centers, including DNA extraction, incubation conditions, and interpretation of the colorimetric reaction, a marked difference in assay sensitivity after 60 min of incubation was observed. This finding raises concerns regarding assay reproducibility, pre-analytical variability, and the external validity of the protocol. In addition to potential technical factors, biological variables such as lesion chronicity, fungal burden, previous antifungal therapy, and differences in the distribution of clinical presentations between study populations may also have contributed to the observed inter-center variability. Additionally, *S. brasiliensis* strains circulating in Rio de Janeiro and Rio Grande do Sul are genetically distinct [21], which may have contributed to the differences in LAMP performance observed between the two centers. Together, these findings emphasize the importance of multicenter validation studies, as diagnostic assays that perform well under controlled laboratory conditions or in single-center evaluations may not maintain comparable performance across different clinical settings.

These findings suggest that, in its current format, this LAMP protocol is not accurate to reliably support the diagnosis of human sporotrichosis in endemic settings. Previous research on LAMP assays for fungal diseases has highlighted the difficulty of achieving consistently high diagnostic performance in clinical practice. For instance, a LAMP protocol evaluated for fungal keratitis was unable to detect all true cases when compared with qPCR, which achieved a sensitivity of 84% in corneal scrapings [22]. Focusing on histoplasmosis, the most prevalent endemic mycosis worldwide, published LAMP assays further illustrate the challenges of translating promising molecular approaches into robust clinical diagnostics. The first assay, targeting the single-copy *Hcp100* gene, demonstrated good analytical specificity under laboratory conditions but showed limited clinical sensitivity, detecting *Histoplasma* DNA in only 67% of antigen-positive urine samples [23].

Several factors may explain the occurrence of false-negative LAMP results in culture-confirmed sporotrichosis cases. These include low fungal burden, uneven distribution of fungal elements within lesions, and the presence of host-derived or microbial inhibitors that interfere with amplification. A meta-analysis of LAMP for human fungal pathogens have reported wide variability in sensitivity and specificity across studies, with pooled diagnostic indicators ranging from low to moderate in some cases, reflecting heterogeneity in targets, sample types, and assay conditions. Most importantly, LAMP technology could be considered as a feasible alternative to current diagnostics when high fungal burden occurs [24]. DNA degradation and the low copy number of the target gene were also proposed as contributors to false-negative results. Notably, calmodulin is also a single-copy gene in *Sporothrix* [25, 26], which may similarly have contributed to the low sensitivity observed in our human samples. Likewise, a second LAMP assay targeting the *Histoplasma* ITS region, evaluated primarily in bone marrow samples from HIV/AIDS patients, achieved only modest sensitivity (around 54%) when culture was used as the reference standard, despite high specificity [27]. These examples coupled to the results herein presented emphasize that, despite the theoretical benefits of rapid isothermal amplification, real-world diagnostic accuracy for human mycoses can be limited and highly dependent on clinical sample quality, pathogen burden, and assay design [28].

False-positive LAMP results were also observed, particularly at longer incubation times, and were noted in patients with other infectious diseases, including tuberculosis and chromoblastomycosis. This finding raises concerns regarding nonspecific amplification or cross-reactivity when reactions are allowed to proceed beyond optimal time points. Colorimetric LAMP assays are known to be susceptible to late nonspecific reactions, pH changes unrelated to target amplification, and subjective interpretation of color shifts, all of which may compromise specificity in field or routine laboratory use [29]. These limitations reinforce the importance of strict time control and cautious interpretation of positive results.

In contrast, LAMP performance in cat samples was comparatively better, particularly at shorter incubation times, with a higher proportion of early positives among culture-confirmed cases, as occurred in other LAMP assay designs from other groups [15, 17]. This divergence between human and cat results highlights important biological and methodological differences that must be considered. Cats with sporotrichosis typically present with lesions characterized by a high fungal burden, and extensive tissue involvement [30], conditions that may favor molecular detection. In humans, especially in fixed cutaneous and lymphocutaneous forms, fungal load is often low as evidenced by a qPCR assay [31], potentially falling below the analytical sensitivity threshold of the LAMP assay when applied directly to clinical samples. This comparative analysis emphasizes that diagnostic tools for sporotrichosis cannot be universally applied across host species without careful validation and adjustment. A One Health approach remains essential, but host-specific factors must be explicitly accounted for in assay design and interpretation [7, 32].

The discrepancy between the favorable results previously obtained in animal models [16] and the limited performance observed in this clinical study underscores a critical issue in diagnostic assay development: results generated under controlled experimental conditions do not necessarily translate to routine clinical practice. In animal models, variables such as inoculum size, lesion age, sampling site, and absence of prior antifungal therapy are tightly controlled [33]. In contrast, real-world clinical samples are inherently heterogeneous, influenced by lesion chronicity, sampling technique, host immune response, and prior treatment, all of which may affect fungal DNA availability and assay performance.

Our findings indicate that, despite its operational advantages, the evaluated LAMP protocol does not currently provide sufficient diagnostic performance for human sporotrichosis in endemic, real-world clinical settings and showed only moderate performance for feline sporotrichosis, with an optimal LAMP incubation time of no more than 40 min. Future improvements may require optimization of DNA extraction methods, refinement of primer design, changes in monitoring methods to fluorometry, or combination with complementary diagnostic approaches. Importantly, this study highlights the value of rigorous clinical validation and transparent reporting of limitations, which are essential steps toward the responsible development of point-of-care diagnostics for neglected fungal diseases.

## Supplementary Information

Below is the link to the electronic supplementary material.

Supplementary Material 1

Supplementary Material 2

## Data Availability

All data that support the findings of this study are included within this paper and its Supplementary Information files.
